# Connections between eating psychopathology, loneliness, and quality of life: insights from a multi-center study

**DOI:** 10.3389/fpsyt.2024.1439179

**Published:** 2024-10-02

**Authors:** Patrizia Todisco, Laura Maragno, Anna Marzotto, Barbara Mezzani, Fabio Conti, Luca Maggi, Paolo Meneguzzo

**Affiliations:** ^1^ Eating Disorder Unit, Casa di Cura “Villa Margherita” – Neomesia, Vicenza, Italy; ^2^ Department of General Psychology, University of Padova, Padova, Italy; ^3^ Eating Disorders Unit, Casa di Cura Villa dei Pini - Neomesia, Firenze, Italy; ^4^ Eating Disorders Unit, Casa di Cura Villa Armonia - Neomesia, Roma, Italy; ^5^ Eating Disorders Unit, Casa di Cura Ville di Nozzano - Neomesia, Lucca, Italy; ^6^ Department of Neuroscience, University of Padova, Padova, Italy; ^7^ Padova Neuroscience Center, University of Padova, Padova, Italy

**Keywords:** quality of life, loneliness, health-related quality of life, eating disorders, anorexia nervosa, bulimia nervosa, binge eating disorder (BED)

## Abstract

**Background:**

Eating disorders (ED) involve dysfunctional attitudes towards food intake, affecting physical and psychosocial well-being. These disorders significantly impact various domains of life and can lead to a decrease in health-related quality of life (HRQoL). Recent studies emphasize the importance of addressing HRQoL in ED treatment, particularly in the context of social isolation and loneliness, but this aspect is currently poorly evaluated.

**Methods:**

A sample of 220 people with an ED was enrolled for the study from different centers specialized in the treatment of ED and compared to 151 people from the general population. Different validated questionnaires were used to evaluate eating psychopathology, HRQoL, and loneliness. Partial correlation analyzes adjusted for marital status and regressions were used to evaluate the relationships between constructs and the differences between groups.

**Results:**

Higher feelings of loneliness were associated with a poorer HRQoL in patients and controls. In the ED group, both loneliness and eating psychopathology were significant predictors of HRQoL. Meanwhile, the duration of the disorder predicted HRQoL specifically in underweight patients, and BMI was a predictor of HRQoL in individuals with normal or above-normal weight.

**Conclusions:**

These findings highlight the importance of considering both HRQoL and loneliness in EDs, particularly among younger individuals. This approach aligns with the increasing focus on the role of interpersonal relationships in the recovery process. Additionally, the data confirm a link between weight and loneliness, suggesting that this connection, especially the differences between underweight patients and those of other weights, deserves further investigation.

## Background

Eating disorders (ED) encompass a wide range of mental and organic pathologies characterized by dysfunctional attitudes toward food intake and body weight and shape, leading to significant changes in physical health and psychosocial functioning among affected individuals (1). This classification includes Anorexia Nervosa (AN), Bulimia Nervosa (BN), Binge eating disorder (BED), and other specified feeding or eating disorders (OSFED) that do not meet the criteria for the former diagnoses and can be considered as an internalizing psychopathological continuum by applying a transdiagnostic cognitive behavior approach (2).

Common features of these disorders include abnormal eating or weight control behaviors, distorted attitudes toward nutrition and body weight and shape, and a pattern of reported negative emotions and feelings of loneliness (3). EDs can severely affect various domains of life, resulting in physical, mental, and social impairment (4). An aspect significantly affected in ED individuals is quality of life (QoL), marked by a comprehensive decline in health-related QoL (HRQoL) compared to the general population (5). While QoL is a broad concept encompassing an individual’s overall well-being—including emotional, social, and environmental factors—the literature has primarily focused on HRQoL, which specifically addresses how an individual’s health status impacts their physical, mental, and social well-being. The decline in HRQoL has been associated with the duration and severity of the disorder, but not with the BMI of the patients (5). Furthermore, HRQoL enhancement has been identified as a specific goal for people with a prolonged duration of the disease, who do not respond to specialized treatments (5, 6). This underscores the pivotal role HRQoL plays in the context of ED and underlines its importance in defining the biopsychosocial perspective of the disorder (7).

HRQoL is a multidimensional construct that encompasses subjective perceptions of physical, psychological, social, and functional aspects of health. It correlates with the severity of ED and overlaps with dimensions related to physical, social, and mental health (8). In enduring EDs, impairments and disabilities in all domains of life contribute further to a decreased HRQoL (9). Factors that contribute to poor HRQoL in patients in the ED include negative psychosocial elements such as stress, pain, high analgesic intake, loneliness, and poor sleep quality. Recent events, such as the COVID-19 pandemic, have highlighted the relationship between social isolation and quality of life. In fact, the pandemic exacerbated social isolation and loneliness in ED patients (10), hindering their access to support networks and, consequently, worsening their symptoms.

Loneliness can be a burden for eating psychopathology (11), and EDs might be a dysfunctional attempt to manage and cope with the absence of close relationships (12). Moreover, loneliness can affect the psychological and physical well-being of ED patients (13). Recognizing the importance of interpersonal relationships in EDs, spending time with friends and family has been found to be a motivating factor in the recovery process for ED patients (14). Given the connection between loneliness, social isolation, EDs, and HRQoL, the literature suggests that interventions targeting HRQoL could be beneficial in ED treatment, potentially leading to improved symptoms (15).

To our knowledge, no study in the literature has investigated the connections between loneliness and HRQOL in EDs, despite both elements being recognized as important. Previous research has examined these elements separately, as though they are unconnected, but evidence from the recent pandemic has highlighted the effects of social isolation on well-being. Therefore, in line with the existing literature, the present research aims to investigate the relationships between loneliness and HTQoL in people with ED and the general population, and whether loneliness directly impacts HRQoL in the clinical sample. Specifically, the hypothesis posits that loneliness plays a distinctive role in the deteriorating quality of life among individuals with EDs, showing a potential target for future studies and interventions.

## Methods

This study employed a cross-sectional design to assess participants within one week of admission to specialized ED treatment. To measure key variables such as eating pathology, health-related quality of life, and loneliness, we utilized a survey bundle comprising standardized questionnaires: the Eating Disorders Examination Questionnaire (EDE-Q) for eating psychopathology, the Italian version of the Health-Related Eating Disorder Quality of Life Questionnaire for HRQoL, and the Revised University of California Los Angeles Loneliness Scale (UCLA) for loneliness.

Participants were 220 individuals recruited from four different national eating disorder clinics at the beginning of inpatient treatment and 151 individuals from the general population as healthy controls (HC). Clinical participants met the diagnostic criteria for the anorexia nervosa subtype (ANr, n = 94, 41.9%), the anorexia nervosa subtype (ANbp, n = 34, 15.2%), bulimia nervosa (BN, n = 36, 16.1%), binge eating disorder (BED, n = 36, 16.1%) or other specified feeding and eating disorders (OSFED, n = 24, 10.7%). ED participants were diagnosed by experienced ED clinicians using the semi-structured clinical interview for the DSM-5 criteria routinely used in clinical practice. The high proportion of AN participants in this sample is attributable to the inpatient setting in which they were recruited.

HC participants were recruited by general announcements on social networks asking volunteers for a clinical evaluation of well-being. The only exclusion criteria applied to the HC participants were the presence of a personal history of any psychiatric conditions, assessed using three specific items in the overall questionnaire requesting previous diagnoses, prior psychiatric or psychological treatment, and current or past use of psychiatric medication usage.

All participants described themselves as cisgender and the majority were white (95.9%).

### Measures

Measures were completed as part of a routine service evaluation within one week from admission for the ED group. In the current study, all three questionnaires presented a good internal consistency index (Cronbach’s α > 0.80).

EDE-Q is a 33-item self-report measure of eating pathology with 7-point responses on the Likert scale (16). EDE-Q is made up of a global score and four subscales: restraint, eating concerns, shape concerns, and weight concerns. A higher score indicates greater severity.

EDQoL is a validated 33-item self-report measure of HRQoL in eating disorder patients (15, 17). It is composed of a total score and five subscales: psychological, physical/cognitive, work/school, financial, and interpersonal. Responses are collected on a 5-point Likert scale (0 = never affecting their quality of life, 4 = always affecting their quality of life), with higher scores indicating lower quality of life.

UCLA is a 20-item scale designed to measure subjective feelings of loneliness as well as feelings of social isolation (18). Responses score from 1 to 4 on a Likert scale, with higher scores indicating elevated levels of loneliness.

A standard demographic and treatment history questionnaire was used to obtain demographic information that was integrated with clinical notes to obtain accurate information about the duration of the disease, weight and height. While for the ED participants the data was collected by professionals, for the HC participants, the data was self-reported.

### Statistical analysis

Descriptive statistics were used to calculate the means and standard deviations of the continuous variables and the percentage and frequency of the categorical variables. Partial bivariate relationships between the severity of symptoms of the ED, HRQoL, and loneliness were examined using Pearson’s correlations controlling for marital status. The comparison between the groups in correlations was performed with an r-to-Z transformation. Linear regression analyses were used to evaluate the possible causal relationships between loneliness, BMI, duration, and severity of the disorders, with HRQoL as the dependent variable. A secondary set of regression analyses was conducted to differentiate between individuals with ED who are underweight and those who are not. Finally, also the predictor value of loneliness for eating psychopathology was tested with regression analysis. The alpha was set at p < 0.05 for all analyses. The analyzes were conducted with IBM SPSS Statistics 25.0 (SPSS, Chicago, IL, United States).

## Results

### Description of the sample

A total sample of 220 people with an ED and 151 individuals from the general population participated in the study. Participants’ ages ranged from 13 to 73 years, their BMI ranged from 11 to 68 kg/m², and their years of education ranged from 8 to 23. We found no significant differences in demographic features between the groups. The descriptive statistics are presented in **Table 1**. No significant differences emerged between individuals with ED and HC in sociodemographic characteristics, except for their current status of relationship. Specifically, most people with ED reported being single (78.2%), while the majority of HC participants reported being engaged but not cohabiting (52.9%). As expected, based on the literature, significant differences emerged in psychological characteristics, with people with ED reporting higher levels of eating psychopathology, lower quality of life, and greater loneliness compared to those without ED.

**Table 1:** Descriptive statistics for the participants.

	ED n = 220	HC n = 151	t	P
Age, y, mean (SD)	26.54 (11.78)	25.88 (6.24)	0.695	0.487
Years of education, mean (SD)	13.40 (2.07)	13.80 (2.44)	-1.730	0.094
Duration of ED, y, mean (SD)	6.99 (8.16)	–	–	–
BMI, mean (SD)	22.18 (11.70)	21.84 (2.75)	0.416	0.677
Gender, female cisgender (%)	200 (90.9)	133 (88,1)	0.780§	0.377
Living condition				
Alone	33	17	6.107§	0.107
Parents	163	121		
Partner	24	9		
Friends	4	4		
Marital status				
Single	172	59	81.370§	< 0.001
Married/Cohabitant	24	12		
Engaged not cohabitant	28	80		
Previous inpatient treatment				
0	117		–	–
1	38	–		
2	19	–		
3 or more	50	–		
EDE-Q Global score, mean (SD)	3.88 (1.51)	1.48 (1.29)	18.347	< 0.001
EDQoL Total score, mean (SD)	68.07 (18.52)	18.32 (15.42)	27.100	< 0.001
UCLA score, mean (SD)	48.26 (12.56)	36.05 (10.29)	10.911	< 0.001

BMI, body mass index; EDE-Q, eating disorder examination questionnaire; EDQoL, eating disorder quality of life; UCLA, university of California Los Angeles loneliness scale. §, Chi-square test.

### Correlations

**Figure 1:**
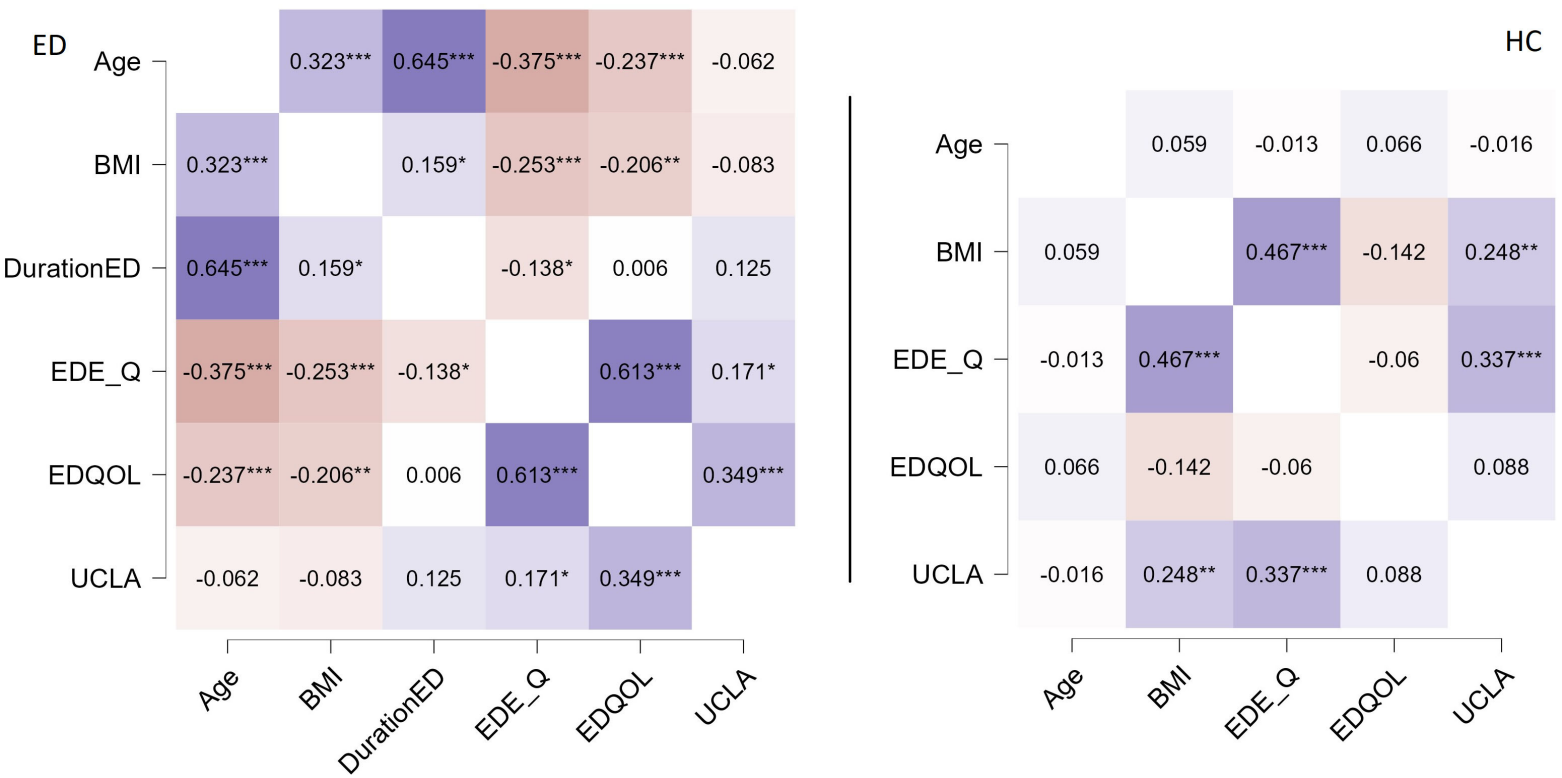
Partial correlation analyses adjusted for marital status. In the left ED group, in the right HC group. Positive correlations are colored purple, while negative correlations are colored in orange. * p < 0.05, ** p < 0.01, *** p < 0.001.

Partial correlation analyzes revealed different relationships between constructs in the two groups. The Fisher r-to-z transformation highlighted specific differences between these correlations. In the HC group, we found significantly different correlations compared to the ED group: a positive relationship between BMI and UCLA, a positive correlation between EDE-Q and UCLA, and a positive correlation between BMI and EDE-Q. In contrast, the ED group exhibited several significant correlations different from those in the HC group: a positive correlation between age and BMI, a negative correlation between age and both EDE-Q and EDQoL, a positive correlation between UCLA and EDQoL, and a positive correlation between EDQoL and EDEQ. Refer to **Table 2** for the Fisher transformation results and **Figure 1** for the partial correlations.

**Table 2:** Fisher’s Z-transformation partial correlation comparison between ED and HC groups.

	Age	BMI	EDE-Q	EDQoL	UCLA
BMI	2.588 0.005	–			
EDE-Q	-3.576 <0.001	-7.174 <0.001	–		
EDQoL	-2.886 0.002	-0.619 0.268	7.258 <0.001	–	
UCLA	-0.376 0.354	-2.817 0.002	-1.689 0.046	2.783 0.003	–

Adjusted for marital status. BMI, body mass index; EDE-Q, eating disorder examination questionnaire; EDQoL, eating disorder quality of life; UCLA, university of California Los Angeles loneliness scale.

### Regression analyses

A series of linear regression analyses were conducted separately for ED patients and the general population to evaluate the possible causal relationships between loneliness, BMI, duration and severity of the disorders, with EDQoL considered as the dependent variable.

For ED patients, the overall regression model was significant, F(4, 208) = 40.44, p <.001, explaining 43% of the variance in EDQoL (adjusted R² = .427). UCLA significantly predicted EDQoL (B = 0.39, SE = 0.08, β = 0.263, t = 4.94, p <.001), indicating that higher levels of loneliness were associated with lower EDQoL. BMI was not a significant predictor of EDQoL (B = -0.08, SE = 0.09, β = -0.50, t = -0.93, p = .353). The duration of the disorder was not significant (B = 0.12, SE = 0.12, β = 0.05, t = 1.01, p = .312), while severity (EDE-Q global score) was a significant predictor (B = 7.63, SE = 0.75, β = 0.56, t = 10.22, p <.001).

In the general population, the regression model was not significant and duration of the disorder was not included: F(3, 132) = 1.61, p = .190, and explained little variance in EDQoL (adjusted R² = .013). None of the predictors—UCLA (B = 0.19, SE = 0.13, β = 0.13, t = 1.46, p = .147), BMI (B = -0.93, SE = 0.56, β = -0.17, t = -1.66, p = .099), or EDE-Q (B = -0.20, SE = 1.19, β = -0.02, t = -0.17, p = .865)—were significant predictors of EDQoL.

Finally, two separate linear regression analyses were conducted within the ED population, one for individuals who were underweight (BMI < 18.5 kg/m²) and another for those with a BMI above 18.5 kg/m². Both models were significant and demonstrated different effects of the duration of the disorders and BMI on EDQoL in different clinical subgroup.

The regression model for the underweight group was significant, F(4, 124) = 25.33, p <.001, explaining 43% of the variance in EDQoL (adjusted R² = .432). Significant predictors in this group included EDE-Q (B = 7.18, SE = 0.91, β = 0.56, t = 7.89, p <.001), UCLA (B = 0.37, SE = 0.11, β = 0.25, t = 3.41, p = .001), and duration (B = 0.36, SE = 0.17, β = 0.15, t = 2.18, p = .031), indicating that greater severity of the disorder, higher levels of loneliness, and longer duration were associated with worse EDQoL.

For individuals with a BMI above 18.5 kg/m², the regression model was also significant, F(4, 79) = 19.29, p <.001, accounting for 47% of the variance in HRQoL (adjusted R² = .469). In this group, significant predictors included EDE-Q (B = 10.58, SE = 1.38, β = 0.70, t = 7.65, p <.001), UCLA (B = 0.43, SE = 0.12, β = 0.30, t = 3.73, p <.001), and BMI (B = 0.29, SE = 0.14, β = 0.19, t = 2.11, p = .038).

Finally, we conducted two separate linear regression analyses to evaluate the predictive value of loneliness on eating pathology, for both the ED group and the general population. In the ED group, the regression model was significant (F(1, 218) = 5.74, p = .017, B = 0.02, SE = 0.01, β = 0.16, t = 2.40, p = .017), indicating that higher levels of loneliness were significantly associated with greater eating pathology. Similarly, in the control group, the regression model was also significant (F(1, 148) = 17.13, p <.001, B = 0.04, SE = 0.01, β = 0.32, t = 4.14, p <.001), suggesting that loneliness was a significant predictor of eating pathology in this group as well.

## Discussion

This study investigated the relationships between loneliness, eating psychopathology, and HRQoL in ED patients compared to the general population. Our findings indicate that loneliness and eating psychopathology uniquely impact HRQoL in individuals with EDs, unlike in the general population. These results might suggest that interventions targeting both loneliness and eating psychopathology could be particularly effective in improving HRQoL for those with EDs, highlighting a need for future research in this area. Several recent studies have reported a reduction in quality of life for individuals with a lifetime diagnosis of ED (19), as well as persistent impairment in various areas of social functioning, even after recovery (20). These elements carry significant psychological and economic burdens (21), as ED treatments are both time-consuming and costly (22).

The lack of associations between the duration of the disorders and HRQoL in our sample has to be considered. It may underscore the persistent negative effects that ED has on people’s lives, regardless of the duration of the disorder. However, for those who are underweight, especially individuals diagnosed with anorexia nervosa, the duration of the disorder may have a more pronounced effect, highlighting potential differences in how various EDs impact HRQoL. The effect of disorder duration on HRQoL is more clearly established in anorexia nervosa compared to other eating disorders, supporting the idea that the impact may vary across different types of EDs (23). In patients who are underweight and have severe, long-term eating disorders, improving HRQoL has been identified as a potential treatment goal (6). Our data also suggest that factors like social inclusion and support are important for enhancing HRQoL. Additionally, higher BMI is associated with lower HRQoL in individuals with bulimia nervosa, binge-eating disorder, and other specified feeding or eating disorders, indicating that increased body weight can negatively affect quality of life (24). The negative correlation between HRQoL and age implies that younger individuals may have unique needs regarding quality of life that should be addressed early on. Future research should focus on comprehensive interventions that target both the psychological aspects of eating disorders and factors such as loneliness, to prevent these issues from worsening and further impacting HRQoL (25, 26).

Loneliness has a connection with dysfunctional eating behaviors in our sample, both clinical and from the general population, corroborating the evidence that emerged during the recent pandemic (27). Our data confirm the presence of positive correlations between loneliness and eating psychopathology and also identify loneliness as a predictor of ED, suggesting that loneliness may contribute to worsening specific psychopathology, potentially due to social exclusion, isolation, and poor connections (28, 29). These data might explain the few reports that identified positive recovery outcomes during pandemic isolation due to the support of families, resulting in a reduction in loneliness (10). Potentially, the relationship identified in our analysis might suggest that interventions targeting loneliness or social isolation could improve eating behaviors, indicating that reducing loneliness may have a positive impact on eating disorders (27, 30, 31). Furthermore, these results are in line with evidence of a relationship between disordered eating and interpersonal dynamics, where social exclusion or overinclusion has a role in acute cognitive response (31). Besides, we found that a higher BMI is related to greater loneliness in the general population, corroborating the idea that a higher body weight might be associated with an increased sense of exclusion (32). This aspect is currently discussed in the literature, and our data support the notion that the connection between loneliness and weight deserves further exploration (13, 33).

Quality of life has been proposed as the primary outcome of treatment in enduring EDs, focusing on the overall impact of the disorder rather than just on its symptoms (34). However, this proposal has several critical limitations (6), and our data could help shed light on these limitations. HRQoL might be impacted by specific psychopathology and loneliness, and both elements require targeted interventions; otherwise, improvements may not be sustainable. In this perspective, social recovery appears to be a potential focus for treatment, possibly allowing modification of the psychopathological core of eating disorders (35, 36).

It is essential to acknowledge the limitations of our study and consider how they may affect our main findings. First, we relied solely on self-report questionnaires, which may introduce response biases that could influence the accuracy of the reported associations. Second, our use of a cross-sectional design limits our ability to determine causal relationships, suggesting that longitudinal studies are needed to better evaluate the directionality and causality of the observed relationships. Lastly, our sample predominantly consisted of white cisgender women, which may limit the generalizability of our findings to more diverse populations. Future studies should also include other factors that might affect HRQoL and response to treatment, such as personality traits, childhood traumatic events, and comorbidities like anxiety or other psychiatric conditions (23, 37, 38).

## Conclusion

In conclusion, this study has explored the relationships between loneliness, eating psychopathology, and HRQoL among individuals with an ED and the general population. The findings suggest that loneliness in people with an ED is strongly related to HRQoL, emphasizing the need for interventions that address both loneliness and HRQoL, rather than addressing only one aspect. Additionally, the study demonstrated a connection between loneliness and weight, including weight concerns, in the general population, highlighting the need for future studies on the effects of perceived exclusion by others.

## Data Availability

The raw data supporting the conclusions of this article will be made available by the authors, without undue reservation.
